# Collimonas rhizosphaerae sp. nov., a novel species isolated from the beech rhizosphere

**DOI:** 10.1099/ijsem.0.006481

**Published:** 2024-07-30

**Authors:** Stephane Uroz, Ségolène Bouche, Emmanuelle Morin, Mathilde Bocquart, Ravi Kumar, Michael W. Rey, Jonathan Pham, Fidel Akum, Johan H. J. Leveau

**Affiliations:** 1 Université de Lorraine, INRAE, UMR1136, Interactions Arbres-Microorganismes, 54000 Nancy, France; 2 INRAE, UR1138, Biogéochimie des Ecosystèmes Forestiers, F-54280 Champenoux, France; 3 Novozymes Inc., 1445 Drew Ave., Davis, CA 95618, USA; 4 Department of Plant Pathology, University of California, Davis, CA 95616, USA

**Keywords:** beech forest soil, *Collimonas rhizosphaerae*, *Oxalobacteraceae*

## Abstract

Bacterial strain H4R21^T^ was isolated from beech rhizosphere soil sampled in the forest experimental site of Montiers (Meuse, France). It effectively weathers minerals, hydrolyses chitin and produces quorum sensing signal molecules. The strain is aerobic and Gram-stain-negative. Phylogenetic analysis based on its 16S rRNA gene sequence indicated that strain H4R21^T^ belongs to the genus *Collimonas* with high sequence similarity to *C. arenae* Ter10^T^ (99.38 %), *C. fungivorans* Ter6^T^(98.97 %), *C. pratensis* Ter91^T^ (98.76 %), *C. humicola* RLT1W51^T^ (98.46 %) and *C. silvisoli* RXD178 ^T^ (98.46 %), but less than 98 % similarity to other strains of the genus *Collimonas*. The predominant quinone in H4R21^T^ is ubiquinone-8 (Q8). The major polar lipids are diphosphatidylglycerol, phosphatidylethanolamine, diphosphatidylglycerol, phosphatidylglycerol and lipid. The major fatty acids identified were C_12 : 0_, C_12:0_ 3-OH, C_16  :  0_ and C_17:0 _cyclo. The digital DNA G+C content of the genomic DNA was 59.5 mol%. Furthermore, the strain could be clearly distinguished from its closely related type strains by a combination of phylogenomic and *in silico* DNA–DNA hybridization results, and phenotypic characteristics. Therefore, strain H4R21^T^ represents a novel species within the genus *Collimonas*, for which the name *Collimonas rhizosphaerae* sp. nov. is proposed, with strain H4R21^T^ (=CFBP 9203^T^=DSM 117599^T^) as the type strain.

## Introduction

The genus *Collimonas* belongs to the family *Oxalobacteraceae* and is considered as a member of the rare biosphere, since metagenomic analyses reveal that its abundance in soil compartments is typically very low [1,5]. Paradoxically, though rarely found in metagenomic studies, *Collimonas* strains are culturable and can be isolated relatively easily [5,7]. In term of ecology, representatives of the genus *Collimonas* are typically found in nutrient-poor and acidic soils with limited human disturbance and usually rich in fungi, suggesting an adaptation to oligotrophic conditions [4,8,12]. Microcosm experiments have validated this hypothesis, showing that even a low input of potassium or magnesium in a soil that is naturally poor in these nutrients induced a fast reduction of the abundance of *Collimonas* [7]. Members of the genus *Collimonas* are Gram-stain negative, aerobic rods and usually motile, although with some exceptions [9,14]. Regarding the functional potential of *Collimonas*, independent screenings of different collections have revealed a conserved ability to hydrolyse chitin and to utilize fungal hyphae as carbon source, to inhibit fungal growth, to weather minerals, and to produce quorum-sensing signal molecules [8,15,21]. The ability to inhibit fungal growth was shown to depend on various molecules (*e.g*. collimonin and carenaemin) [18,19]. Noticeably, some *Collimonas* have been shown to promote plant growth [20]. *Collimonas* represent an interesting genus with promising properties, which invites deeper analyses into their taxonomy and functional abilities. Currently, six *Collimonas* species names have been validly published, namely *C. anthrihumi*, *C. arenae*, *C. fungivorans*, *C. pratensis*, *C. humicola* and *C. silvisoli*) [9,12].

In this study, we performed a polyphasic taxonomic and functional characterization of strain H4R21^T^. Our analyses provide phenotypic, genomic and phylogenomic evidence that this strain meets the criteria for delineating a new species of *Collimonas*. Strain H4R21^T^ is of special interest as it is able to metabolize fungal derivates (*e.g*., chitin, trehalose, mannitol), to solubilize inorganic phosphorus and complex minerals (e.g., biotite, garnet, apatite) [22], to inhibit fungal growth and to produce quorum-sensing signal molecules.

## Isolation and ecology

Strain H4R21^T^ is part of a collection of 370 bacterial strains that was established during a study on the functional abilities of bacterial isolates from beech rhizosphere and their comparison with the properties of bacteria from bulk soil [15]. Sampling was done on the forest experimental site of Montiers (48.53 N, 5.32E) which is located in the Meuse department (in northeastern France) under mono-specific plots of beech trees. After removal of the litter layer, cores (33×33 cm, and 5–20 cm deep, representing the organo-mineral soil horizon) were collected. From the rhizosphere soil of one of these cores, strain H4R21^T^ was isolated by serial dilutions on 1/10 diluted tryptic soy agar (TSA) medium [Difco’s tryptic soy broth (TSB) 3 g l^−1^ and agar 15 g l^−1^] with a pH adjusted to 6.5. The isolate was purified by three successive spreads on the same medium to obtain a pure culture. Strain H4R21^T^ was grown routinely on 1/10 TSA (or 1/10 TSB) or on low salt LB (LBm) medium for 3 days at 25 °C, under agitation (150 r.p.m.) for liquid media. Its purity was routinely confirmed by macro- and microscopic observations, by chitin degradation assay (description below) and by sequencing of its 16S rRNA gene and its genome. Stock cultures were stored at −80 °C in LBm medium plus 40 % glycerol. Chemical characteristics of the soil samples from which strain H4R21^T^ was isolated were as follows: pH 4.63±0.04, 17.20±0.74 g kg^−1^ total carbon, 1.19±0.07 g kg^−1^ total nitrogen, 0.38±0.13 g kg^−1^phosphorus extracted according to the Duchaufour method and 29.78±13.92 g kg^−1^ organic matter [15].

Strain H4R21^T^ (DSM 117599^T^=CFBP 9203^T^) is available in the following public culture collections: Deutsche Sammlung von Mikroorganismen und Zellkulturen (DSMZ; http://www.dsmz.de/collection) and the CIRM-CFBP (Collection française des bactéries associées aux plantes). Reference strains *C. humicola* RLT1W51^T^ (KACC 21985^T^), *C. silvisoli* RXD178^T^ (KACC 21987^T^), *C. antrihumi* C3-17^T^ (DSM 104040^T^), *C. arenae* Ter10^T^ (DSM 21398^T^), *C. pratensis* Ter91^T^ (DSM 21399^T^), *C. fungivorans* Ter 6^T^ (DSM 17622^T^) and Ter331, as well as *C. arenae* Ter282, *Collimonas* sp. Cal35 and *C. pratensis* PMB3(1) were obtained from lab collections, the DSMZ or from the Korean Agricultural Culture Collection (KACC) and included in our analyses for comparison to H4R21^T^.

## 16S rRNA gene phylogeny

Genomic DNA of strain H4R21^T^ was extracted according to Pospiech and Neumann [23] after a sequential treatment with: (i) lysozyme (1 mg ml^−1^) at 37 °C for 1 h, (ii) addition of 1/10 of sodium dodecyl sulphate (final concentration, 1 %) and (iii) proteinase K (final concentration, 1 mg ml^−1^). DNA was recovered after chloroform purification and isopropanol precipitation. Full-length 16S rRNA gene sequences were recovered from isolate H4R21^T^ and all reference strains, and aligned in Seaview with muscle [24]. A phylogenetic tree was reconstructed using the PhyML algorithm with *Caballeronia mineralivorans* PML1(12)^T^ as outgroup.

**Fig. 1.**
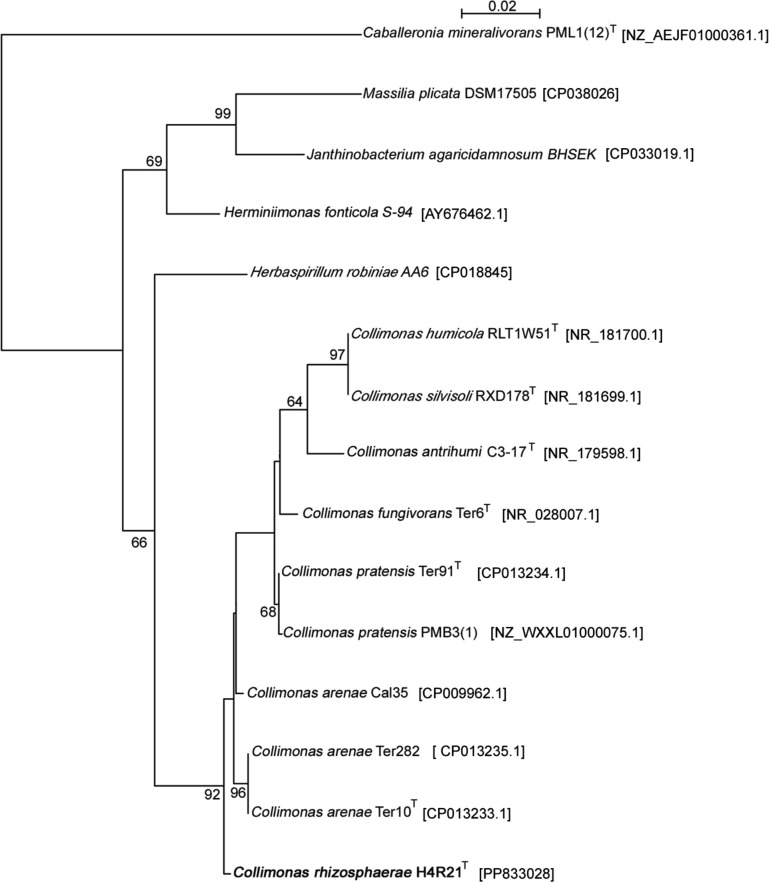
Maximum-likelihood (PhyML) 16S rRNA gene phylogenetic tree based on 16S rRNA gene sequences showing the relationship between strain H4R21^T^ and other closely related genera and species in the family *Oxalobacteraceae*. Bootstrap values are expressed as a percentage of 1000 replications. Only branch-support values ≥50 % are included.

The 16S rRNA gene sequence comparisons and phylogenetic tree analyses confirmed that strain H4R21^T^ is a member of the genus *Collimonas*. Comparative analysis of the 16S rRNA gene sequence of strain H4R21^T^ (accession number: PP833028PP833028) with those of other *Collimonas* type species revealed that this strain is closely related to species *C. areneae* Ter10^T^ (99.38 %), *C. fungivorans* Ter6^T^ (98.97 %), *C. pratensis* Ter91^T^ (98.76 %), *C. humicola* RLT1W51^T^ and *C. silvisoli* RXD178^T^ (both 98.46 %), and had less than 98 % similarity to other members of the genus *Collimonas*. However, the phylogenetic tree revealed that this new strain forms a phylogenetic branch that is separated from all the other published type strains of the genus, as suggested by the bootstrap value of 92 % (Fig. 1). Strain H4R21^T^ is also separated from the other *Collimonas*-related genera (*Herbaspirillum*, *Janthinobacterium*, *Massilia*, *Herminiimonas*).

## Core gene-based phylogenomic analyses

The whole genome of strain H4R21^T^ was sequenced as 2×150 bp paired reads on an Illumina NextSeq 500 instrument (accession numbers of whole-genome sequence and raw sequences: GCA_038896935.1 and JBANDC010000000GCA_038896935.1 and JBANDC010000000). The assembly was generated with SPAdes version 3.9.0 [25]. The draft genome is 5.6 Mb and is composed of a total of 46 contigs larger than 500 bp. Similar genome sizes have been reported for other species of *Collimonas* (5.7 Mb for *C. pratensis* Ter91^T^, 5.6 Mb for *C. fungivorans* Ter6^T^), while a smaller genome size of 4.7 Mb was observed for *C. arenae* Ter10^T^ (and Ter282) and *C. antrihumi* C3-17^T^ (Table 1) [11,14]. No plasmid feature was detected, as was the case in the genomes of *C. fungivorans* Ter331 and *C. arenae* Cal35. The genome of strain H4R21^T^ has a G+C content of 59.52 mol%, similar to the G+C contents reported for *C. silvisoli* RXD178^T^ (59.45 mol%) and *C. humicola* RLT1W51^T^ (59.4 mol%), but higher than other species (54.6 mol% for *C. antrihumi* C3-17^T^ and 58.99 mol% for *C. fungivorans* Ter6^T^) (Table 1) [11,14].

**Table 1.** Differential biochemical characteristics of all examined strains of species of the genus *Collimonas* Strains: 1, C. *rhizosphaerae* H4R21^T^; 2, *C. antrihumi* C3-17^T^; 3, *C. humicola* RLT1W51^T^; 4, *C. silvisoli* RXD178^T^; 5, *C. arenae* Ter10^T^; 6, *C. arenae* Ter282; 7, *C. arenae* Cal35; 8, *C. fungivorans* Ter6^T^; 9, *C. pratensis* Ter91^T^; 10, *C. pratensis* PMB3(1). Differential biochemical characteristics of all examined strains of species of the genus. +, Positive; −, negative; w, positive with a weak signal. All Biolog, chitin hydrolysis and iron chelating data were generated in this study. The data presented for temperature, pH and NaCl ranges of the reference bacterial strains come from the literature.

Characteristics	1	2	3	4	5	6	7	8	9	10
**Genomic features**										
Size (Mb)	5.6	4.72	5.63	5.66	4.7	4.7	5.6	5.6	5.7	5.6
G+C content (mol%)	59.52	54.6	59.4	59.45	57	57	56	58.99	58.5	59
**Physiological and functional features**										
Temperature range for growth (15–30 °C)	+	+	+	+	+	+	+	+	+	+
pH range for growth (pH 5–8)	+	+	+	+	+	+	+	+	+	+
NaCl range for growth (0.5–2 %)	+	+	+	+	+	+	+	+	+	+
Chitin hydrolysis	+	−	+	w	w	w	+	+	w	+
Chelating activity	+	−	+	+	+	+	+	+	+	+
**Utiliation of substrate (Biolog**)										
2-Aminoethanol	+	−	−	+	−	−	−	+	−	−
α-Cyclodextrin	−	−	−	−	−	−	−	+	−	−
α-d-Glycerol-1-phosphate	−	−	−	+	−	−	−	−	−	−
α-d-Lactose	−	−	−	−	−	−	−	+	−	−
α-Hydroxybutyric acid	−	+	+	+	+	+	−	+	−	−
α-Ketobutyric acid	+	−	+	+	+	+	−	+	−	−
α-Ketovaleric acid	−	−	+	+	−	−	−	−	−	−
Acetic acid	−	−	+	+	−	+	−	+	−	−
Adonitol	−	−	−	−	−	−	+	+	−	−
Methyl β-d-glucoside	−	−	−	−	−	+	−	−	−	−
Bromosuccinic acid	+	+	+	+	+	+	+	+	−	+
d-Galacturonic acid	−	−	−	−	−	−	+	+	−	−
d-Gluconic acid	+	+	+	+	+	+	−	+	+	+
d-Glucosaminic acid	+	+	+	+	+	+	−	+	+	+
d-Glucose-6-phosphate	+	+	+	+	+	+	+	−	+	+
d-Psicose	+	−	+	−	+	+	−	+	−	−
d-Raffinose	−	−	−	−	−	−	−	+	−	−
d-Sorbitol	+	+	+	−	−	−	+	+	+	+
Trehalose	+	+	−	+	−	−	−	+	+	+
d-Alanine	+	−	+	+	+	−	−	−	−	−
d-Arabitol	+	−	+	+	+	+	+	+	+	+
Cellobiose	−	−	−	+	−	−	−	−	−	−
d-Galactonic acid lactone	+	−	+	+	+	+	−	+	+	+
d-Mannitol	+	−	+	−	+	+	+	+	+	+
d-Mannose	+	−	+	+	+	+	+	+	+	+
Melibiose	−	+	−	−	−	−	−	−	−	−
d-Serine	−	−	−	+	−	−	−	−	−	−
d,l-α-Glycerol phosphate	+	+	+	+	+	+	−	+	+	+
d,l-Carnitine	+	−	−	−	−	−	−	−	−	−
Dextrine	+	+	+	+	+	+	−	+	+	−
γ-Hydroxybutyric acid	−	−	+	−	−	−	−	−	+	−
Gentibiose	−	+	−	−	−	−	−	−	−	−
Glucuronamide	+	−	+	+	+	+	+	+	+	+
Glycerol	+	−	+	+	+	+	−	+	+	+
Glycogen	+	−	+	+	+	−	−	−	−	−
Hydroxy-l-proline	−	+	+	+	−	−	+	−	+	+
i-Erythritol	−	−	−	−	−	−	−	+	−	−
Inosine	+	−	+	+	+	+	−	+	+	+
l-Alanine	+	−	+	+	+	+	+	+	+	−
l-Histidine	+	+	+	+	+	+	+	+	−	+
l-Leucine	+	+	+	+	+	+	−	+	+	+
l-Ornithine	+	−	+	−	+	+	−	+	−	−
l-Phenylalanine	−	+	+	+	−	−	−	−	−	−
l-Rhamnose	−	−	−	−	−	−	−	+	−	−
l-Threonine	+	−	+	+	+	+	+	+	+	−
Lactulose	−	+	−	−	−	−	−	+	+	−
Malonic acid	+	−	−	+	−	−	−	+	−	−
Maltose	−	−	−	+	−	−	−	+	−	−
*N*-Acetyl-d-galactosamine	−	−	−	−	−	−	+	−	−	−
*p*-Hydroxyphenylacetic acid	−	+	−	−	−	−	−	−	−	−
Phenylethylamine	+	+	−	−	−	−	−	+	−	−
Quinic acid	+	+	−	+	+	+	+	+	+	−
Succinic acid monomethyl ester	−	+	+	+	+	+	−	+	+	+
Sucrose	−	+	−	+	−	−	−	−	−	−
Turanose	−	+	−	+	−	−	−	−	−	−
Uridine	+	−	+	+	+	+	+	+	+	+
Urocanic acid	−	−	+	+	−	−	+	+	+	+
Xylitol	+	−	+	−	−	−	+	+	+	+

All the following substrates are metabolized by all the *Collimonas* strains tested: (α-d-glucose, α-ketoglutaric acid, β-hydroxybutyric acid, *cis*-aconitic acid, citric acid, d-glucoronic acid, d-fructose, d-galactose, d-saccharic acid, d,l-lactic acid, formic acid, γ-aminobutyric acid, glycyl-l-aspartic acid, glycyl-l-glutamic acid, l-alainamide, l-allanyl-glycine, l-arabinose, l-asparagine, l-aspartic acid,L-fucose, l-glutamic acid, l-proline,L-pyroglutamic, l-serine, *m*-inositol, *N*-acetyl-d-glucosamine, propionic acid, pyruvic acid methyl ester, succinamic acid, succinic acid, tTween 40, and tTween 80). All the following substrates are not metabolized by all the *Collimonas* strains tested: (2,3 butanediol, itaconic acid, putrescine, sebacic acid, and thymidine).

**Fig. 2.**
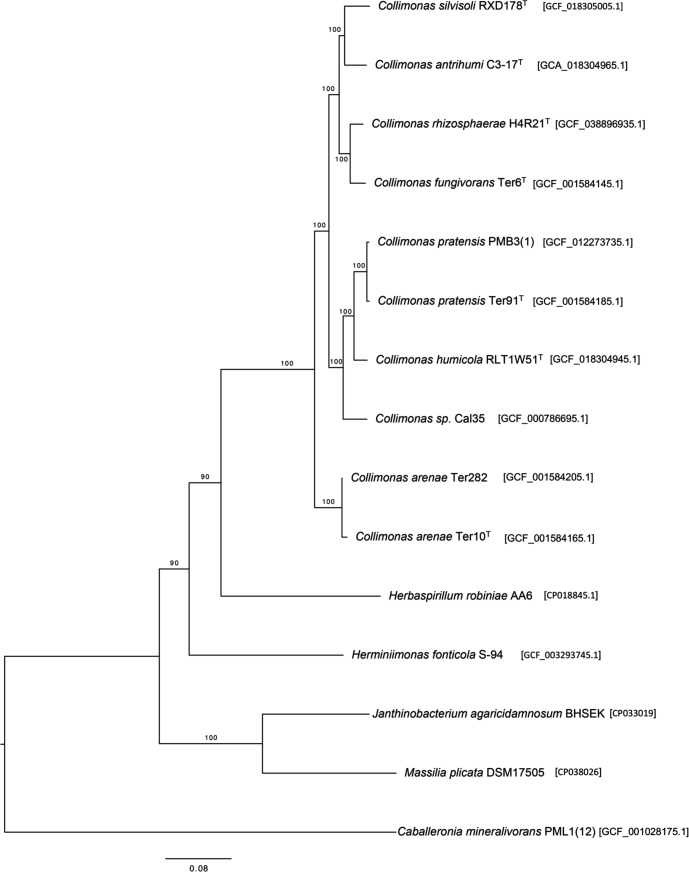
busco marker-based phylogenetic tree showing the relationship between strain H4R21^T^ and other closely related genera and species in the family *Oxalobacteraceae*. The phylogenetic tree was inferred using 527 single-copy genes. Bootstrap values are expressed as a percentage of 1000 replications.

The assembled genomes of other *Collimonas* strains were obtained from the GenBank database for comparison to the genome of strain H4R21^T^. TYGS [26] was used to generate the average nucleotide identity (ANI) and digital DNA–DNA hybridization (dDDH) relatedness based on genome sequences. busco analysis (version 5.6.1 [27]) showed a genome completeness at 99.9 % as compared to the *Burkholderiales* odb10 lineage data set. According to the busco marker-based phylogenetic tree that was inferred using 527 single-copy genes with RAxML-NG (version 0.9.0) [28] applying a partitioned analysis and 1000 bootstraps replicates, strain H4R21^T^ clustered closest with *C. fungivorans* Ter6^T^*, C. antrihumi* C3-17^T^ and *C. silvisoli* RXD178^T^ (Fig. 2). ANI values between strain H4R21^T^ and the closely related type species were calculated as ranging between 83.79 and 90.82 %. Correspondingly, the dDDH values ranged from 35.5 % with *C. arenea* Cal35 to 60.2 % with *C. fungivorans* Ter6^T^(Table 1). Considering the recognized threshold values (ANI 95‒96 % and dDDH 70 %), strain H4R21^T^ represents a novel *Collimonas* species.

## Morphology and physiology

Cell morphology and motility were observed with a BX41 Olympus microscope at a magnification of ×1000 and by using phase contrast and differential interference contrast with unstained living bacteria. A Gram characterization was performed using the aminopeptidase test from Sigma. For all the assays described below, the bacterial inoculum was prepared as follows. Bacterial strains were grown for 2 days in liquid LBm, centrifuged at 7000 *g* and washed twice with sterile milliQ water. The optical density (OD=595 nm) of each bacterial suspension was adjusted to 0.9 (corresponding to ca. 1×10^8^ c.f.u. ml^−1^). Antibiotic susceptibility was tested in liquid LBm with ampicillin, chloramphenicol, kanamycin, streptomycin (all at final concentration: 100, 150 and 200 µg ml^−1^), gentamicin (final concentration: 20 and 50 µg ml^−1^) and tetracycline (final concentration: 10, 20 and 50 µg ml^−1^). Motility was tested on M9 mineral medium (20 mM NH_4_Cl; 12 mM Na_2_HPO_4_; 22 mM KH_2_PO_4_; 8.6 mM NaCl; 1 mM MgSO_4_; 1 mM CaCl_2_; 0.2 % glucose; and 0.5 % casamino acids) amended with 0.25 % agar, following the recommendation of Turnbull and Whitchurch [29]. A volume of 1 µl bacterial inoculum was spotted on the centre of the agar in the Petri dish and colony diameters were scored after 2 days. Growth at different temperatures (15, 20, 25, 30 and 35 °C) was assessed in LBm medium after incubation for 10 days. The pH range for growth (pH 3.0–10.0, at intervals of 1.0 pH unit) was also tested in LBm medium, as was tolerance of increasing NaCl concentrations (0.5–6 %, with an interval of 1 %, w/v). For all these assays, growth was determined by measuring OD at 595 nm with a spectrophotometer (Bio-Rad, iMark). The ability to hydrolyse chitin was tested on minimal agar medium containing colloidal chitin (per litre: 5 g NaCl, 1 g KH_2_PO_4_, 0.1 g yeast extract, 20 g agar, and 2 g colloidal chitin) [21]. After incubation at 25 °C for 14 days, the clearing of the initially turbid medium indicated chitin hydrolysis and the diameters of the resulting halos were measured. The production of *N*-acyl homoserine lactone (AHL) was assessed in T-streak assays [30] with the *Chromobacterium violaceum* CV026 biosensor [31]. The production of violacein was scored after 2 and 4 days of incubation. The siderophore activity was performed according to Schwyn and Neilands [32], after a 2 day incubation in liquid AB medium (devoid of iron) amended with citrate as sole carbon source (2 g l^−1^), by mixing a 1 : 1 volume of supernatant and a volume of chromazurol S (CAS) solution. After 1 h of incubation at 20 °C, the OD was measured at 655 nm with a spectrophotometer (Bio-Rad, iMark). The ability to utilize different carbon substrates was determined for each bacterial strain using the GN2 microplates (Biolog), which permits simultaneous testing of 95 substrates. OD values were recorded at 595 nm and considered positive ‘+’ for OD values greater that 0.1 and negative ‘0’ for OD values lower than 0.1.

Cells of strain H4R21^T^ are non-spore-forming and shaped rods (approx. 2–3 µm long and 1 µm wide). The different assays performed revealed that strain H4R21^T^ is aerobic, Gram-negative and motile, and susceptible to chloramphenicol, gentamicin, kanamycin, streptomycin and tetracycline, but resistant to ampicillin (up to 200 µg ml^−1^). The observation of motility was consistent with the presence of genes encoding flagellar biosynthesis and assembly as well as the type IV pili system in the genome of H4R21^T^. Noticeably, flagellar biosynthesis and assembly encoding genes were detected in the genomes of the motile strains of *C. fungivorans*, *C. arenae* and *C. pratensis*, but not in the genomes of the non-motile *C. antrihumi*, *C. silvisoli* and *C. humicola*. Colonies of strain H4R21^T^ are transparent, circular and off-white with a circular margin around the main colony on 1/10 TSA incubated for 3 days at 25 °C. Growth in LBm liquid medium occurred at 15–30 °C (optimum at 20 °C), pH 5–8 (optimum at pH 5) and with 0.5–2 % NaCl. The chitin degradation assay revealed that strain H4R21^T^ is able to hydrolyse chitin to the same extent as *C. fungivorans* Ter6^T^ and *C. pratensis* PMB3(1), but better than *C. arenae* Ter10^T^ and *C. pratensis* Ter91^T^, while no hydrolysis was observed for the reference strains of *C. antrihumi*, *C. silvisoli* and *C. humicola*. The siderophore production assay revealed that strain H4R21^T^ is able to chelate iron as well as other tested strains (*C. fungivorans*, *C. arenae*, *C. pratensis*, *C. silvisoli* and *C. humicola*)*,* except *C. antrihumi* C3-17^T^. The production of AHL was demonstrated for strain H4R21^T^as well as for strains of *C. fungivorans* (Ter6^T^, Ter331) and *C. pratensis* [Ter91^T^ and PMB3(1)], for *C. arenae* (Ter282), *Collimonas* sp. strain Ca35, * C. silvisoli* (RXD178^T^) and *C. humicola* (RLT1W51^T^), but not for *C. antrihumi* [C3-17^T^] and *C. arenae* (Ter10^T^). Homologues of AHL synthase (*i*.*e*., *luxI*-like) and of the cognate transcriptional regulator LuxR were detected in the genomes of all AHL-producing strains, including H4R21^T^. Noticeably, strain H4R21^T^ is able to consume d-mannitol, trehalose and *N*-acetyl-d-glucosamine, which are all known as fungal metabolites. d-Mannitol was not assimilated by *C. antrihumi* (C3-17^T^) and *C. silvisoli* (RXD178^T^) and trehalose not by *C. humicola* (RLT1W51^T^), *C. arenae* (Ter10^T^, Ter282) and *Collimonas* sp. strain Cal35. All other physiological and metabolic properties of H4R21^T^ and *Collimonas* strains are shown in Table 2.

**Table 2.** Average nucleotide identity (ANI) and digital DNA–DNA hybridization (dDDH) relatedness (%) based on genome sequences

ANI score										
Strain	Cal35	C3-17^T^	H4R21^T^	PMB3(1)	RLT1W51^T^	RXD178^T^	Ter10^T^	Ter282	Ter6^T^	Ter91^T^
Cal35	100	83.34	83.80	85.16	85.49	83.48	82.63	82.63	83.73	85.05
C3-17^T^	83.31	100	85.89	83.87	84.20	87.05	83.17	83.21	85.77	83.86
**H4R21^T^**	**83.79**	**85.93**	**100**	**85.86**	**86.23**	**86.94**	**83.83**	**83.88**	**90.82**	**85.79**
PMB3(1)	85.15	83.94	85.91	100	90.22	84.36	83.73	83.79	86.33	98.17
RLT1W51^T^	85.39	84.26	86.29	90.11	100	84.81	83.84	83.85	86.45	90.09
RXD178^T^	83.37	87.08	86.90	84.37	84.85	100	83.32	83.37	86.60	84.40
Ter10^T^	82.47	83.16	83.83	83.64	83.77	83.39	100	99.97	83.75	83.68
Ter282	82.48	83.20	83.80	83.67	83.79	83.39	99.97	100	83.75	83.73
Ter6^T^	83.72	85.81	90.82	86.14	86.31	86.67	83.71	83.78	100	86.27
Ter91^T^	85.02	83.95	85.74	98.14	90.18	84.37	83.72	83.74	86.34	100

ddH scores										
Strain	Cal35	C3-17^T^	H4R21^T^	PMB3(1)	RLT1W51^T^	RXD178^T^	Ter10^T^	Ter282	Ter6^T^	Ter91^T^
Cal35	100	30	35.5	43.6	47.6	32.5	29.9	30	34.7	43.1
C3-17^T^	30	100	37.4	32.4	34.7	40.9	32.3	32.4	36.9	32.2
**H4R21^T^**	**35.5**	**37.4**	**100**	**43.8**	**43.9**	**45.8**	**36.9**	**37**	**60.2**	**43.8**
PMB3(1)	43.6	32.4	43.8	100	57.1	33.4	33.8	33.8	46.2	78.3
RLT1W51^T^	47.6	34.7	43.9	57.1	100	36.5	35.2	35.3	41.5	55.7
RXD178^T^	32.5	40.9	45.8	33.4	36.5	100	32.9	32.9	41.5	34.1
Ter10^T^	29.9	32.3	36.9	33.8	35.2	32.9	100	100	34.1	33.5
Ter282	30	32.4	37	33.8	35.3	32.9	100	100	34.2	33.6
Ter6^T^	34.7	36.9	60.2	46.2	41.5	41.5	34.1	34.2	100	47.9
Ter91^T^	43.1	32.2	43.8	78.3	55.7	34.1	33.5	33.6	47.9	100

## Chemotaxonomy

Cellular fatty acids of strain H4R21^T^ were analysed by the DSMZ (Braunschweig, Germany) using biomass grown for 48 h on LBm at 25◦C. Fatty acids were analysed as methyl esters by gas chromatography according to the instructions of the Microbial Identification System (midi Inc., USA) as described by Tindall [33,34] and Kuykendall *et al*. [35].

The major respiratory quinone identified for strain H4R21^T^ is ubiquinone 8 (Q-8), which is also found in the other *Collimonas* species [11,12]. The polar lipid analyses revealed that strain H4R21^T^ is characterized by phosphatidylethanolamine, diphosphatidylglycerol, phosphatidylglycerol, aminophospholipid, glycolipid, aminolipid, phospholipid and lipid. Diphosphatidylglycerol was also present in *C. humicola* and *C. antrihumi*, and variable among *C. fungivorans* strains and in *C. silvisoli* [11,12] (Fig. S1 in the online Supplementary Material).

The major fatty acids (>5 % of the total) of strain H4R21^T^ were C_16  :  0_ (35.3 %), C_17:0 _ cyclo (6.6 %), summed feature 3 (C_16 : 1_* ω*6*c* and/or C_16 : 1_ ω7*c*; 35 %) and summed feature 8 (C_18 : 1_* ω*7*c* and/or C_18 : 1_* ω*6*c*; 11.8 %) (Table 3). C_16  :  0_ was detected within the expected range of concentrations (33–40 %) observed for the reference *Collimonas* strains. Noticeably, strain H4R21^T^ had a lower relative abundance of C_17:0_ cyclo (6.6 % in strain H4R21^T^ *vs* 13–24 % in other *Collimonas*) and a higher relative abundance of summed feature 3 (35 % in strain H4R21^T^ vs 9.6–22.6 % in other *Collimonas*) [11,12], making this strain distinguishable from the other *Collimonas* species.

**Table 3.** Cellular fatty acid profiles of strain H4R21^T^ and closely related type strains of the genus *Collimonas* Strains: 1, *C. rhizosphaerae* H4R21^T^; 2, *C. fungivorans* Ter6^T^; 3, *C. arenae* Ter10^T^; 4, *C. pratensis* Ter91^T^; 5, *C. antrihumi* C3-17^T^; 6, *C. silvisoli* RXD178^T^; 7, *C. humicola* RLT1W51^T^. Data are expressed as percentages of the total fatty acids. The major components (>10% of the total) are highlighted in bold. nd, not detected. The data presented for the reference bacterial strains come from the literature.

Fatty acid	1	2	3	4	5	6	7
C_10:0_ 3-OH	0.6	0.9	1.0	0.9	1.1	1.1	0.8
C_12 : 0_	4.9	3.4	4.1	3.4	3.6	3.4	3.8
C_12:0_ 2-OH	1.4	2.5	1.9	2.4	2.5	2.2	2.0
C_12:0_ 3-OH	2.1	3.1	3.4	3.7	3.8	3.3	3.3
C_14 : 0_	0.8	nd	nd	nd	nd	nd	nd
C_16 : 0_	35.3	40.2	40.8	40.2	33.4	35.6	38.5
C_16:1_ 2-OH	nd	1.4	1.9	2.4	4.4	2.5	2.5
C_17:0_ cyclo	6.6	24.1	17.0	21.5	13.1	19.4	21.7
iso-C_17:0_ 3-OH	nd	2.8	nd	2.3	1.9	2.4	2.5
C_18 : 0_	1.2	1.3	1.7	0.7	1.5	1.6	nd
Summed feature 3*	35	15.4	9.6	18.8	22.6	12.7	14.2
Summed feature 8*	11.8	4.5	6.8	7.2	9.6	13.2	7.6

*Summed features are fatty acids that cannot be resolved reliably from another fatty acid using the chromatographic conditions chosen. The midi system groups these fatty acids together as one feature with a single percentage of the total. Summed feature 3, C_16:1_ω7*c* and/or C_16:1_ω7*t*; summed feature 8, C_18:1_ω7*c* and/or C_18:1_ω7*t*.

## Description of *Collimonas rhizosphaerae* sp. nov.

*Collimonas rhizosphaerae* (rhi.zo.sphae’rae. Gr. fem. n. *rhiza*, a root; Gr. n. *sphaira*, a ball, a sphere; N.L. fem. n. *rhizosphaera*, the rhizosphere; N.L. gen. fem. n. *rhizosphaerae*, of the rhizosphere, referring to the source of isolation of the type strain).

Colonies on 1/10 TSA medium are transparent, circular and slightly white with a differentiation of a circular margin around the main colony. Growth requires 3 days on LBm, 1/10 TSA and ABm media. No pigment is observed on these media. Tolerates up to 2 % NaCl and grows at temperatures between 15 and 30 °C, with an optimum temperature of 20 °C. Growth occurs at pH 5–8 and the optimum is pH 6. Positive for the hydrolysis of colloidal chitin and siderophore production. The following carbon sources are used: 2-aminoethanol, α-d-glucose, α-ketobutyric acid, α-ketoglutaric acid, β-hydroxybutyric acid, bromosuccinic acid, *cis*-aconitic acid, citric acid, d-gluconic acid, d-glucuronic acid, d-glucosaminic acid, d-glucose-6-phosphate, d-psicose, d-sorbitol, trehalose, d-alanine, d-arabitol, d-fructose, d-galactonic acid lactone, d-galactose, d-mannitol, d-mannose, d-saccharic acid, dl-α-glycerol phosphate, dl-carnitine, dl-lactic acid, dextrine, formic acid, γ-aminobutyric acid, glucuronamide, glycerol, glycogen, glycyl-l-aspartic acid, glycyl-l-glutamic acid, inosine, l-alanine, l-alainamide, l-allanyl-Glycine, l-arabinose, l-asparagine, l-aspartic acid, l-fucose, l-glutamic acid, l-histidine, l-leucine, l-ornithine, l-proline, l-pyroglutamic, l-serine, l-threonine, *m*-inositol, malonic acid, *N*-acetyl-d-glucosamine, phenylethylamine, propionic acid, pyruvic acid methyl ester, quinic acid, succinamic acid, succinic acid, Tween 40, Tween 80, uridine and xylitol. In contrast, strain H4R21^T^ is unable to assimilate: 2,3 butanediol, α-cyclodextrin, α-d-glycerol-1-phosphate, lactose, α-hydroxybutyric acid, α-ketovaleric acid, acetic acid, adonitol, methyl β-d-glucoside, d-galacturonic acid, raffinose, cellobiose, melibiose, d-serine, γ-hydroxybutyric acid, gentibiose, hydroxy-l-proline, *i*-erythritol, itaconic acid, l-phenylalanine, l-rhamnose, lactulose, maltose, *N*-acetyl-d-galactosamine, *p*-hydroxyphenylacetic acid, putrescine, sebacic acid, succinic acid monomethyl ester, sucrose, thymidine, turanose and urocanic acid are not assimilated. The predominant quinone is ubiquinone-8 (Q8). The major polar lipids are diphosphatidylglycerol, phosphatidylethanolamine, diphosphatidylglycerol, phosphatidylglycerol and lipid. Major fatty acids (>10 %) are C_12 : 0_, C_12:0_ 3-OH, C_16 : 0_ and C_17:0_ cyclo.

The type strain is H4R21^T^ (=DSM 117599^T^= CFBP 9203^T^), isolated from beech rhizosphere collected from the forest experimental site of Montiers (Meuse, France). The whole genome shotgun project has been deposited at DDBJ/ENA/GenBank under the accession number JBANDC000000000. Raw data is available from the SRA database with accession number PRJNA1081282.

## supplementary material

10.1099/ijsem.0.006481 Fig. S1.

## Abbreviations

ANI: average nucleotide identity
dDDH: digital DNA-DNA hybridization
