# Supplementary material for: Collimonas rhizosphaerae sp. nov., a novel species isolated from the beech rhizosphere
Source: Int J Syst Evol Microbiol. 2024 Jul 30;74(7):006481. doi: 10.1099/ijsem.0.006481 (PMC11288634; doi:10.1099/ijsem.0.006481)
Supplement: Fig. S1. [file ijsem-74-06481-s001.pdf]

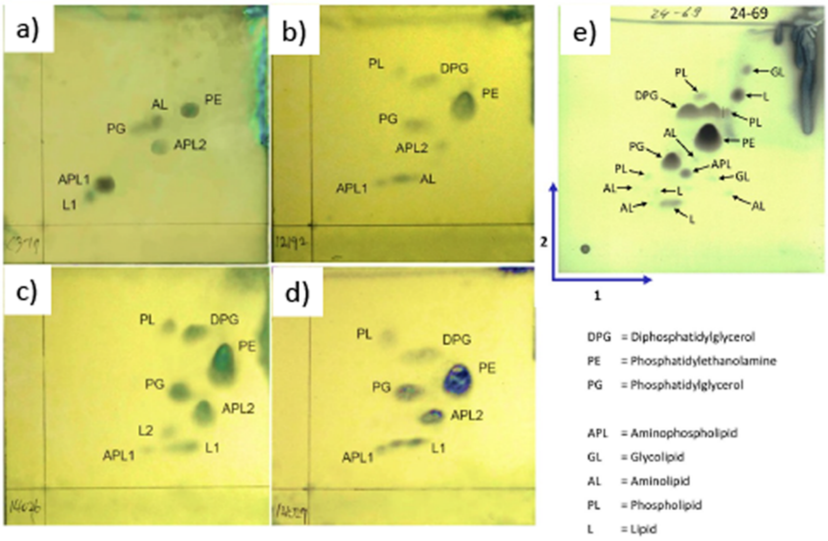

Figure S1. Polar lipid profile of the different types strains and of the strain H4R21^{T}. a) *C. antrihumi* C3-17^{T} ; b) *C. fungivorans* Ter6^{T}; c) *C. arenae* Ter10^{T}; d) *C. pratensis* Ter91^{T} (a to d, adapted from Li *et al.*, 2021); e) *C. rhizosphaerae* H4R21^{T} (from this study).
